# When Clinical Trials Are Compromised: A Perspective from a Patient Advocate

**DOI:** 10.1371/journal.pmed.0020358

**Published:** 2005-10-18

**Authors:** Musa Mayer

## Abstract

In her personal essay, Mayer argues that many trained patient advocates are just as concerned as health professionals are with getting the very best evidence from clinical trials.

Twelve years ago, a friend from my breast cancer support group went to court because her insurance company had refused coverage for a bone marrow transplant. Her first transplant had failed and her cancer was progressing again. The insurance company refused coverage for the second transplant on the basis that it was an experimental treatment. The judge, a cancer survivor himself, was clearly moved by her appeal, and my friend got her transplant. Six months later, she was dead—not from her metastatic breast cancer, but from treatment-induced damage to her bone marrow.

Then, a second friend with breast cancer died following her transplant a few months after that, and I began to read the research for myself and to piece together what the studies actually showed—and what they didn't show. My education about clinical trials had begun, as I have previously described in a 2003 essay entitled, “From Access to Evidence: An Advocate's Journey” [1].

It took me some time, and a lot of study, to understand the dynamics of what had actually happened in America with bone marrow transplants in breast cancer. And how wishful thinking on the part of patients and oncologists, public pressure, heart-wrenching media stories of desperately ill young mothers, political and legislative mandates for insurance coverage, personal reputations of researchers, and profit margins of hospitals with transplant beds to fill all managed to widely promote a toxic and expensive treatment before there was sufficient evidence of its safety or efficacy.

## The Rush to Embrace an Unproven Treatment

Hindsight being what it is, we can appreciate the dynamics now, and see how the uncritical adoption of this treatment off trial added years to the time that it took to enroll individuals in the randomized trials that ultimately would answer the question of efficacy. By the end of the decade, in fact, more than 20,000 American women had endured this treatment for no compelling reason. Many died because of it, while others were left with serious and long-lasting side effects. [Fig pmed-0020358-g001]

**Figure pmed-0020358-g001:**
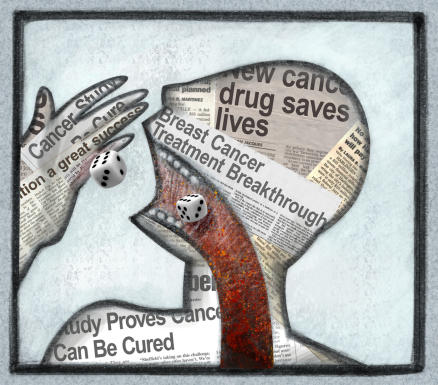
The rush to embrace unproven treatments can end up harming patients (Illustration: Giovanni Maki)

Of course these women were very ill to begin with, and the prevailing wisdom of the time was that desperate circumstances called for desperate measures. Giving doses of chemotherapy so high that the bone marrow was destroyed, then rescuing the patient with her own stem cells or bone marrow—this treatment had intuitive drama and appeal. Many women at the time, including both of my friends mentioned in the introduction, vowed to “go out fighting,” rather than have the longer life and gentler death that might have been theirs with conventional treatment. “If I die,” young women would frequently say, “I want my children to know I did everything I could.” One transplant unit actually used this coercive argument as a marketing ploy.

Naively, I believed until then that doctors could be trusted to rely on good evidence, especially for a treatment as toxic and costly as this one. Certainly, they would never allow themselves to be misled by partial evidence or a compelling theory—that more is better, or that dramatic tumor response in uncontrolled Phase II trials of the high-dose regimens actually predicted for clinical benefit. Or, even more shocking, that one existing small randomized trial that many questioned as flawed—and which later, in fact, turned out to have been falsified—would be held up to patients as good evidence for the treatment [2–5].

My education about clinical trials had begun.

Looking back now, I can trace my radicalization as a patient advocate, and my interest in the proper conduct of randomized clinical trials, to the troubling discovery that in the case of bone marrow transplant in patients with breast cancer, the tools of science had been subverted by the rush to embrace an unproven treatment. The fact that this could happen was profoundly disillusioning. I was disappointed with oncologists, but more disturbing to me was the role that many advocates had played in guaranteeing broad access to bone marrow transplants, effectively sabotaging enrollment in the randomized trials that would have provided a definitive answer years sooner, saving many lives and much personal suffering, not to mention huge financial expenses.

Three years ago, I recounted this story at the Annual Advocacy Training Conference of the National Breast Cancer Coalition. Since the bone marrow transplant stampede ended in 1999, many women diagnosed with breast cancer more recently were unaware of what had happened during the 1990s, and that the new mantra of “targeted therapy” had only recently replaced the “more is better” model.

The transplant debacle also stiffened the resolve and long-time commitment to evidence-based medicine and research standards held by the National Breast Cancer Coalition (NBCC), a grassroots lobbying and advocacy-training organization committed to the eradication of breast cancer. Standing alone among breast cancer organizations, NBCC had refused to fight for access to a treatment that was still unproven. Their position paper on bone marrow transplant was perceived by many as rigid and uncaring. Yet NBCC's unwavering commitment to the evidence and to the need for trials prior to widespread adoption of the treatment ultimately won them the respect they deserved.

## What I Learned about Clinical Trials

Tragedies can sometimes be instructive. As an advocate, I learned a memorable lesson about how clinical trials can go terribly awry through the premature adoption of an unproven therapy. This extraordinarily painful example taught me—and many breast cancer advocates—a great deal about clinical trials: the limitations of Phase II studies, the crucial role of randomization and control groups, the perils of selection bias and stage migration, and surrogate endpoints, such as tumor response, that fail to predict clinical benefit. I also learned how incredibly important it is to preserve the integrity of clinical trials for patients now and in the future. It is a matter of life and death.

In the years since, the conduct of randomized clinical trials has often been in jeopardy. What prompted me to recount this dark chapter in our history to the NBCC advocates were the current legal activities of an organization known as the Abigail Alliance (http://abigail-alliance.org). Founded by surviving family members of patients with cancer who had been unable to get access to experimental treatments under development, with support by antiregulatory forces in Washington, D.C., Abigail Alliance first brought a citizen's petition and then a lawsuit against the United States Food and Drug Administration (FDA). They claimed that current restrictions on experimental treatments represented an infringement on the civil rights of dying patients. They proposed a regulation permitting the marketing of experimental treatments after Phase I trials to patients who had no other treatment alternatives, claiming that this would in no way interfere with the conduct of confirmatory trials.

They were firmly convinced that their loved ones could have been saved, if only they had been permitted access. To them—as to me a decade earlier, before I understood what was at stake—the benefit from these cutting-edge treatments was obvious. The need was urgent. People they loved were dying. New treatments had been developed. How could anyone be cruel enough to deny a patient the next new treatment that might save or extend life? Randomized trials were seen as not only unnecessary but ethically indefensible. To them, the notion of equipoise was simply an absurdity. Strong perceptions of drug efficacy, nurtured by pharmaceutical industry advertising, kept hope alive.

At first, the Abigail Alliance initiative to market drugs after Phase I trials seemed so absurd that many of us advocates didn't take it seriously, and took no action. But the alliance was very serious and very determined. Publicized with the smiling face of their founder's deceased daughter, Abigail, this group acquired considerable media attention, appearing on NBC's *Today Show* and inspiring a *Wall Street Journal* editorial with the memorable title, “FDA to Patients: Drop Dead” [6].

Of course, the first wave of activism for early access to treatments had come from AIDS advocates, giving rise to “accelerated approval,” or Subpart H regulations, in 1993, which permitted drugs to reach the market early in the case of life-threatening illnesses for which no other treatment existed (see http://www.accessdata.fda.gov/scripts/cder/onctools/Accel.cfm). These approvals could be based on surrogate endpoints in uncontrolled trials, with the provision that clinical benefit must be ultimately shown in post-marketing randomized, controlled studies. In the intervening years, many cancer drugs have been approved in this way.

Meanwhile my own understanding of issues in clinical trials continued to evolve. Since my work focuses on women with metastatic breast cancer, my keen interest in drug development and clinical research led to my becoming a Patient Representative and Consultant in the FDA's Cancer Drug Development Program.

## Accelerated Approval of Cancer Drugs

In September 2002, the Oncologic Drugs Advisory Committee recommended accelerated approval of AstraZeneca's drug gefitinib (Iressa) based on a 10% tumor response rate in late-stage non-small-cell lung cancer [7–9], despite concurrent negative findings in large randomized controlled trials [10,11]. It was a heated, emotional meeting, with many patients who otherwise would not have been alive offering personal testimony of benefit from the drug. Obviously, some drug effect was present in this small minority of patients. Many others present however, were disturbed by the precedent set by the vote for approval, with the actual evidence showing tumor response in only 20 patients in two small Phase II trials. Other people wondered why no target had been found for this “targeted” therapy to better predict response and nonresponse, as it had for trastuzumab (Herceptin) and hormonal therapies in breast cancer.

The FDA held an Oncologic Drugs Advisory Committee meeting the following spring, at which we reviewed seven cancer drugs for eight indications that had been granted accelerated approval, but had failed to complete the confirmatory trials. Avoiding the problem that many drugs given accelerated approval had had enrolling individuals in their trials once the drug was on the market, AstraZeneca agreed to complete its confirmatory trial of gefitinib overseas. But ultimately, gefitinib failed to show a benefit in the large mandatory confirmatory “Iressa survival evaluation in lung cancer” trial [12–15].

Trained evidence-based advocates should have a seat and a voice at every table where clinical trials are designed and implemented.

Meanwhile, independent researchers had managed to identify the epidermal growth factor receptor mutation that selects for most of the 10% of patients with lung cancer who respond to the drug [16–18]. Then in November 2004, Genentech's competing epidermal growth factor receptor inhibitor, erlotinib (Tarceva), secured full FDA approval. In the face of the failed confirmatory trial for gefitinib, FDA effectively removed the drug from the market, while allowing patients already responding to gefitinib to continue with their treatment. Among many other issues, the story of gefitinib in lung cancer illustrates the pressing need for concurrent development of biomarkers that select for treatment response to targeted therapies.

Early access to treatments and the impact on clinical trials is of course only one of the many important issues with clinical trials that could be addressed, but I've emphasized it here because it represents an arena that has engaged the patients and the public so consistently during my years as an advocate.

## Early Closure of Trials

Earlier this year, I spoke at the annual meeting of the American Society for Clinical Oncology on a related issue—the ethical and clinical dilemmas relating to the early closure of clinical trials in breast cancer (a recording of the talk, together with the slides I presented, is available at http://asco.org/ac/1,1003,_12-002511-00_18-0034-00_19-001290-00_21-001,00.asp). Such early closure has occurred with increasing frequency in recent years, notably in the P-1 breast cancer tamoxifen prevention trial [19], the MA-17 trial of letrozole (Femara) after tamoxifen [20], and most recently, the adjuvant trastuzumab (Herceptin) trials [21].

The issue of early trial closure is similar to that of accelerated approval of an experimental drug—in both cases, the balance of immediate needs for patients being treated today must be weighed against the knowledge gained that will advance evidence-based medicine and help patients in the future. Patients facing treatment decisions in the future, after mature results of clinical trials have been published, clearly benefit most from the completion of well-designed randomized trials with meaningful endpoints and long periods of follow-up. Their needs are rarely served by stopping clinical trials early, or by trial designs that do not randomize trial participants, examine toxicity carefully, look at overall survival, or follow-up with patients to pick up any unanticipated late-term effects.

## Evidence-Based Patient Advocacy

It has been important for us as advocates to speak out on these issues in every available forum, as individuals and as organizations. Speaking out in this way educates the public as well as the medical and research communities. In my 2003 essay [1], I defined “access advocates” as those who see their role as arguing, as Abigail Alliance does, for earliest access, regardless of the affect on clinical research.

Everyone requires evidence-based care.

When I wrote that 2003 essay, I wanted health professionals to know, as I want you to know today, that the perception of advocates clamoring for early access and compromising clinical trials is far from a complete picture. Many trained advocates are just as concerned as health professionals are with getting the very best evidence from clinical trials. We can help. Our stories have the power to move the public, to influence policy and legislation, and to help enroll patients in trials that they will want to be part of. I believe trained evidence-based advocates should have a seat and a voice at every table where clinical trials are designed and implemented. Together with scientists and clinicians, we can help health professionals to define the most meaningful questions, and ensure that the design and conduct of trials are everything they should be. And we can help to educate the public about the need for well-designed, properly implemented clinical trials.

As a writer, I understand the power of stories. Stories humanize policy, and offer the personal context in which policies and positions actually matter to people. Without our human stories to illustrate and elucidate cause and meaning, the positions health professionals take will not be very meaningful to the public and to the patients they hope to enroll in clinical trials. Properly told, stories have the power to move people, to change minds and hearts. Potentially, they have the power to reach a public who has little understanding of the research enterprise, and barely grasps the need for clinical trials. Everyone is touched by illness. Everyone requires evidence-based health care. I think we need to stop allowing the public dialogue on clinical research to be controlled by the drug companies and by mass media. We need to tell these important stories and express our strong convictions.

My work as an advocate and my personal experience with NBCC tells me that policy positions are important, and that we can have an influence if we are willing to stand up for our principles. An organization, like an individual, is known by the positions it takes and the values it holds. Consistent, well-reasoned evidence-based positions command respect, if not always agreement. So does steadfast refusal to take the expedient position, even when it may be more popular. These are the hallmarks of what can only be called integrity.

*This Essay was adapted from a talk delivered at the Annual Meeting of the Society for Clinical Trials, in Portland, Oregon, May 2005.*

## Abbreviations

FDA: Food and Drug Administration
NBCC: National Breast Cancer Coalition
